# A Behavioral Perspective for Improving Exercise Adherence

**DOI:** 10.1186/s40798-024-00714-8

**Published:** 2024-05-20

**Authors:** Nathalie André, Marine Grousset, Michel Audiffren

**Affiliations:** 1 Research Centre on Cognition and Learning, CNRS, University of Poitiers, Poitiers, France; 2 Richelieu Centre, French Red Cross Association, La Rochelle, France; 3 Maison des Sciences de l’Homme et de la Société, CNRS, University of Poitiers, Poitiers, France

**Keywords:** Behavioral change, Willpower, Motivation, Attitude change, Prediction models of behavior

## Abstract

Adherence to exercise is a crucial aspect of behavioral changes related to exercise. However, current models fail to predict behavioral change, and exercise programs struggle to foster adherence. In this Current Opinion article, we defined adherence as a process linking behavior and attitude. More specifically, exercise adherence is the process by which people’s behaviors conform to their attitudes and vice versa. Behavioral change theories should be able to predict changes, transformations, and modifications of behaviors; however, this is not currently the case. Prediction models of behavior are mainly focused on how to predict behavioral changes by affecting attitudes; however, these models have not considered the target behavior as a facilitator of adherence. Herein, a behavioral perspective is considered in three directions: first, exercise is a therapeutic modality that has the potential to improve motivation; second, chronic exercise helps sustain effort over time and increase executive functions and willpower; and third, exercise is an active behavior that has the potential to facilitate attitude change.

## Key Points

**•** Behavioral change techniques should take into account processes involved during exercise or processes for long-term change.
**•** Attitude change can be facilitated by committing people to their exercise behaviors.
**•** The repetition of a short-term cycle linking exercise to effort-based decision-making leads to long-term changes in the strength of the link between attitude and behavior.

## (Re)Defining Exercise Adherence

Adherence is a construct involved in behavioral change; therefore, the processes underlying behavioral change require consideration. These processes have been well elucidated in the literature and mainly target variables associated with attitudes and intentions [1–6]. Among all the attitudinal determinants of behavior, self-efficacy, intention, perceived barriers and benefits are the best predictors of exercise or physical activity [7–14]. However, an important part of the variance in behavioral change is not explained by these determinants [15, 16]. One possible explanation is that models that aim to predict behavioral change are actually behavior prediction models. Contrary to their stated aims, these models do not focus on behavioral change but rather on established behaviors [17]. There is a substantial difference between what accounts for or explains established behavior and what enables and explains behavioral change. Behavioral change theories should be able to predict behavioral changes, transformations, and modifications; however, this is not presently the case. Favoring change could involve focusing on behaviors first (rather than on attitudes). Because attitudes take time to change, considering behavior (e.g., exercising) as another possible source of change might be an effective intervention for improving exercise adherence.

Adherence is classically defined as an agreement or an approval, and this has naturally led to adherence and compliance being used interchangeably [18, 19]. This definition of adherence, however, leads to a weakened meaning of the term. Adherence is also defined as the “state of a thing strongly attached to another” or a “force which opposes the separation of two [entities] brought into contact”. In the literature on adherence (i.e., medicine, health psychology, exercise psychology), this attachment is generally defined as the strength of the link between the patient and the prescriber. Herein, we will assume an alternative view by defining adherence as the strength of the link between attitudes and behavior. In other words, adherence can be defined as the process by which people behave as they think or think as they behave. From that perspective, exercise adherence is the process by which people’s attitudes conform to their behavior and vice versa. This perspective can be applied in all contexts in which exercise adherence takes place, such as rehabilitation, exercise reconditioning, performance-based physical therapy, and training.

A behavioral-oriented perspective is lacking in classical models of behavioral change, and we suggest considering exercise (i.e., the target behavior) as an important determinant of behavioral change and adherence. In the following sections, we will show that exercise can improve adherence (the strength of the link between attitude and exercise behavior) in three ways. First, exercise is a therapeutic approach with a variety of modalities (e.g., individually or in group, exergames or more conventional exercises, outdoors or indoors) that have the potential to increase motivation. Second, exercise helps individuals maintain effort over time and has the potential to increase executive functions and willpower. Third, exercise is an effort-based behavior that has the potential to facilitate attitude change.

## Immediate Purposes of Exercise have the Potential to Enhance Motivation

High drop-out rates are generally observed in exercise-based interventions [e.g., 20, 21]. From the perspective of cost‒benefit theories, this finding could be explained by a reluctance of the person to invest effort due to the inherent costs of effort (e.g., fatigue, pain) as well as other possible costs, such as time and money, that outweigh the long-term benefits (e.g., weight loss, improved physical condition, etc.). However, the strength of the link between attitudes and behaviors could be optimized by identifying the short-term or immediate purposes of exercise [22, 23]. In other words, exercising generates immediate purposes that can become additional motivations. Several examples of such immediate purposes will be given in the following paragraph.

Lee et al. [22] proposed a model based on an evolutionary perspective of why people are reluctant to expend energy in physical exercise. The authors describe an exercise-affect-adherence pathway to explain how negative affective responses to exercise are a manifestation of an evolved tendency to avoid energy expenditure that serves no immediate adaptive function. Put another way, exercise can be viewed as an unnecessary energy expenditure behavior because it does not achieve immediate goals. According to the authors, encouraging individuals to involve in regular physical exercise requires identifying the immediate purpose rather than the long-term effects, which are often a source of demotivation. The immediate purposes associated with exercise include: (a) feeling of an increase in energy due to an increase in arousal, (b) satisfaction with achieving meaningful goals, such as setting a personal record, (c) pleasant physical sensations, such as warmth, (d) increases in positive mood, and (e) social interactions with peers.

Behavioral change techniques (BCTs) can function as active ingredients in interventions that facilitate the achievement of immediate goals [24]. Several reviews of BCTs in physical exercise interventions have highlighted the effectiveness of short-term techniques in different populations. For example, Finne et al. [25] and Carey et al. [26] demonstrated that BCTs, such as the use of cues and incentives, rewards (social and nonspecific), or graded tasks, increase motivation to exercise among cancer patients. Another review carried out by Howlett et al. [27] showed that biofeedback, demonstration of behavior, behavior practice/rehearsal, and graded tasks were effective short-term intervention techniques for healthy inactive participants. Other populations, such as people with overweight [28], substance use disorders [29], or dementia [30], also show greater involvement in physical activity when BCT-based interventions target immediate purposes. Thus, to increase motivation during exercise sessions, it is necessary to select the best technique according to the population of interest.

In a similar vein, a recent systematic review on behavioral change domains carried out by Michaelsen and Esch [31] led them to develop a resource model of behavioral change based on the functional mechanisms of BCTs, which include facilitating, boosting and nudging mechanisms. Facilitating and nudging are two mechanisms that can increase motivation during exercise sessions. For example, nudging (using cues and prompts) is a context-dependent strategy intended to involve people in behavioral change (e.g., removing chairs in a gymnasium or displaying images of peers doing physical activity). Facilitating (using knowledge, environmental context or social influences) targets external resources to enable new behavior (e.g., providing social support by using walking groups or developing public fitness trails).

Another way to target the immediate purpose of exercise is by using digital tools such as connected watches. These devices represent an interesting solution for providing different types of information to people while carrying out an exercise session. For example, some models offer a connected watch equipped with a screen allowing real-time information to be displayed (number of steps taken, energy expenditure, instantaneous heart rate) [e.g., 32]. Other models propose the association of a connected watch with artificial intelligence to guide patients in their daily practice of PA, such as achieving task objectives or proposing new tasks [e.g., 33]. Therefore, stimulating and encouraging health behavioral change can be increased by targeting techniques or digital tools that enhance immediate purpose (or goals or benefits), but these are short-term effects [e.g., 34].

In summary, focusing on the immediate purpose of exercise can increase motivation and influence effort-based decision-making in favor of exercise. Exercise adherence is influenced by awareness of the benefits of the action during its execution. Nevertheless, increasing motivation at the level of a single exercise session may not be sufficient to facilitate adherence to exercise. Indeed, repetition of exercise-based decision-making may facilitate regular exercise by increasing adherence.

## Exercising Helps Maintain Effort Over Time

Making the decision to exercise regularly is the result of effort-based decision making [23, 35]. Obtaining benefits from regular exercise requires a person’s involvement in physical activity once a day or several times a week on a regular basis. Effort is therefore associated with both physical effort (i.e., to perform the activity itself) and mental effort (i.e., to maintain involvement over time).

**Fig. 1:**
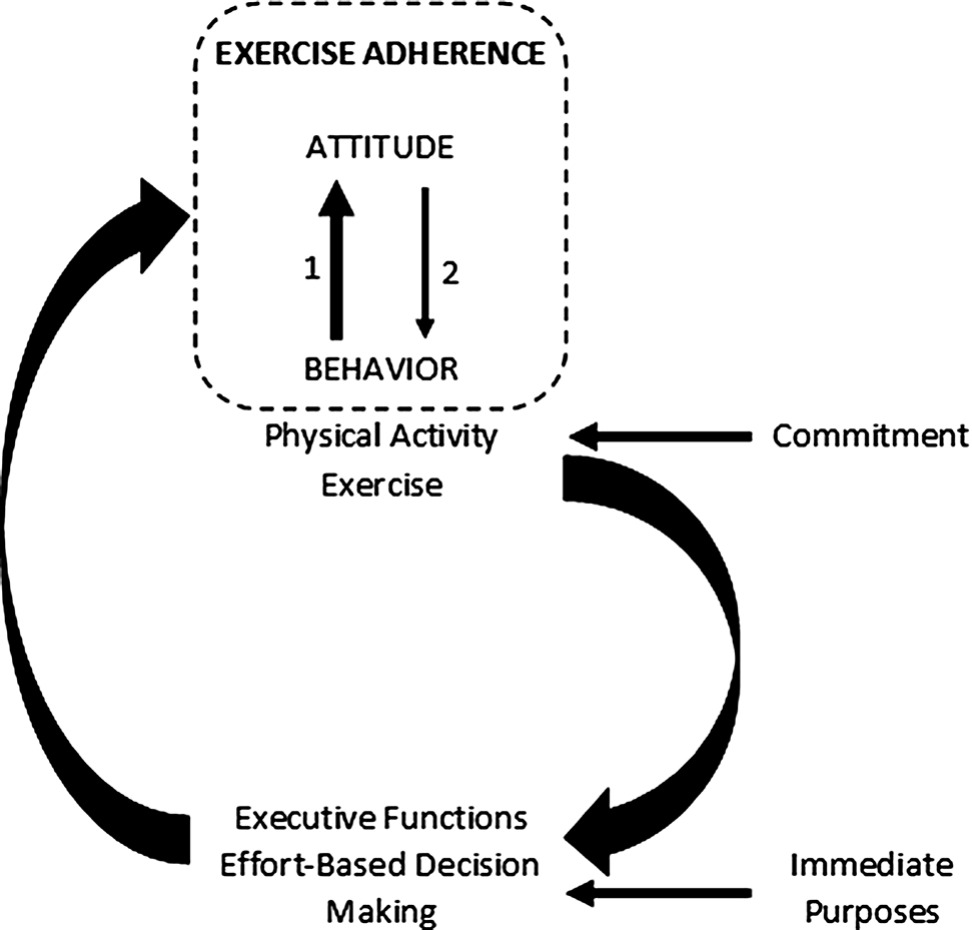
A schematic representation illustrating how behavior can improve exercise adherence. The dark arrows indicate a positive relationship between the variables. The starting point of the cycle is effort-based decision-making, which leads individuals to initiate a behavior. This decision can be influenced by immediate purposes related to exercise, such as increase in arousal and progress. This short cycle happens in the present moment but can be repeated. The repetition of this short cycle will lead to long-term and structural changes (i.e., at the level of the muscle, cardiovascular system, and brain), such as increases in muscular strength, heart-rate variability, self-efficacy, effort value, executive control and willpower, and decrease in effort costs (e.g., fatigue or pain). These changes also include changes in cognitive representations related to exercise (i.e., attitudes) and a strengthening of the links between these attitudes and the behavior, reinforcing adherence to exercise. Commitment to behavior can be induced at the moment of decision-making or once the behavior is initiated

Several authors have proposed that improving executive functioning facilitates adherence to exercise [e.g., 36, 37, 5]. Specifically, it has been shown that exercising regularly can improve the desire to exercise by initiating a virtuous circle. According to the exercise-cognition model of adherence based on evidence from the literature [38], executive functioning (EF) plays a central role in the bidirectional relationship linking exercise and health behavior: chronic exercise leads to an improvement in executive functioning, and improved executive functioning leads to easier adherence to exercise over time. Figure 1 describes the bidirectional relationship between behavior (i.e., exercise) and the cognitive processes that underlie the triggering and maintenance of this behavior (i.e., effort-based decision making, executive functions and attitudes). For example, exercising regularly increases people’s ability to inhibit bad habits, such as staying at home rather than going for a walk, and the positive effects of chronic exercise, such as improved quality of life, increase the value that people will attribute to exercise (or effort). Relatedly, a recent review by Audiffren et al. [39] on training willpower suggested that regular exercise decreases effort costs and increases the value of effort. These mechanisms are involved in exercise adherence.

Among useful executive functions, planning has received much attention. Thus, when effort and increased perseverance are needed, planning helps alleviate willpower issues that are essential for achieving goals [40], for instance, overcoming barriers to exercise or managing pain and discomfort [41]. These plans should facilitate implementation in the face of challenges [42–44]. These beneficial effects of planning are achieved, for instance, through the formation of implementation intentions such as planning how, where and when people will add steps to their daily routine to meet their step goal by using personalized schedules or specific events (e.g., after work). The automatic control involved in implementation intentions is created by a voluntary act rather than established over time via repeated pairings of stimuli and responses. Another possibility is to use BCTs that can increase long-term effort involvement. Goal settings, problem solving, information concerning the consequences for health, and self-monitoring are efficient techniques for promoting willpower [e.g., 25–27, 45]. For example, by monitoring information concerning activities (heart rate during exercise, number of steps performed, level of perceived effort), people can observe the progress made over time.

Similarly, previous studies on exercise habit formation have shown that if exercise cannot be fully automatic, the decision to be active can have the hallmark of automaticity [46]. Thus, it has been shown that time-based cues require monitoring and are, in principle, less suited to supporting action outside awareness than event-based cues, which are inherently more salient and stimulus-driven [47]. For example, a study of preexisting physical activity habits revealed that the consistency of prior events (e.g., ‘after breakfast’) was related to habit strength, but engaging in activity at a consistent time of day was not related to habit strength [48, 49].

Therefore, exerting effort on a regular basis constitutes an effective lever for increasing executive control. In turn, the increase in executive functions facilitates control over– and valuation of– effort. Nevertheless, the gap between involvement in exercise sessions and long-term involvement in exercise can be difficult to bridge. A behavior-based solution might be to commit people to their behavior.

## Exercising can Facilitate Attitude Change

Adherence requires that attitudes be consistent with behavior. However, changing attitudes can be a lengthy process [50] and can slow behavioral change, stop behavioral change, or cause the process of behavior change to be aversive. In contrast, exercising is an active behavior that can be performed instantly. This behavior does not necessarily have to be associated with conformity attitudes to be performed. For example, some people can consider exercise to be a waste of time but agree to carry out a session under the supervision of a counselor. However, they are unlikely to start exercising again unless the counselor has committed them to the behavior first. The low rate of exercise adherence frequently observed among people involved in exercise intervention [e.g.,^51^] may be due to a lack of consistency between attitudes and behavior (i.e., a low fit of attitude to behavior).

Several models from social psychology, such as cognitive dissonance theory [52] and commitment theory [53–55], could be useful for developing new ways of studying adherence to exercise. Indeed, numerous studies based on these models have shown that individuals who are committed to a behavior modify their attitudes *a posteriori* to make them more in line with their past behaviors. Thus, behavioral changes can promote attitude changes through cognitive dissonance [52]. Cognitive dissonance has been studied using several paradigms, including the effort justification paradigm, the difficult decision paradigm, and the induced compliance paradigm, all of which are designed to commit people to their behavior [56]. Since exercising is an effort-based decision, particular attention can be given to the place of effort in these models and how deploying effort in certain circumstances can further commit individuals in their behavior and lead to attitude change. Previous literature has demonstrated that the amount of energy devoted to achieving a goal can play a role in developing or changing an individual’s attitude toward that goal, thereby increasing its value [e.g., 57, 58]. Indeed, people are motivated to place more value on a goal that requires more effort to achieve for at least two reasons. (1) Effort justification is a way of reducing cognitive dissonance by enhancing the value of rewards when they are more difficult to obtain. Assigning greater value to rewards provides justification for the greater effort needed to obtain them [e.g., 59, 56]. (2) Effort heuristic– the belief that effortful actions lead to better outcomes– leads people to expect greater emotional feelings (e.g., happiness) and higher performance quality after high effort rather than after low effort [60]. The positive emotions derived from current actions or anticipated for future experiences play an important role in how people decide whether to engage in effortful activities. This happens because anticipated emotions motivate people to initiate or to persist in goal-seeking behaviors [e.g. 61, 62].

Another paradigm identified in the dissonance literature is the induced-compliance paradigm. Specifically, the “free will” compliance paradigm proposes that the first action is carried out in specific commitment conditions and takes the name of commitment through actions [or preparatory acts, 53–55]. Indeed, after a person makes a decision (i.e., the preparatory act), the negative aspects of the chosen alternative and the positive aspects of the rejected alternative are dissonant with the decision. On the other hand, each of the positive aspects of the chosen alternative and the negative aspects of the rejected alternative are consistent with the decision. Difficult decisions should arouse more dissonance than easy decisions because there will be a greater proportion of dissonant cognitions after a difficult decision than after an easy one. Therefore, one important aspect is that the more committed to an act a person is, the more the individual will change his or her attitudes in the direction of the behavior he or she has performed. Committing conditions could include the context of freedom in which the action was carried out (e.g., to freely agree to sponsor a participant), the repetition of the action (e.g., to deploy the behavior several times a day or a week), the cost of the action (e.g., to have the choice between several levels of effort to successfully perform the behavior) or the reasons for the action (e.g., to attribute the behavior to internal reasons such as seeking pleasure or satisfaction or a better health) [for more explanation, see 54, 55].

Therefore, adherence to exercise can be improved by committing people to their active behavior. This commitment, in return, will favor and strengthen attitude change by reducing dissonance. It is the targeted behavior (i.e., exercise) and the hallmarks of the surroundings (e.g., free will, repetition, cost of effort) that commit people to their behavior and become a source of change. Here, attitudes change as behavior becomes established, thereby reinforcing adherence.

## Discussion

Exercise behavior can be a crucial determinant of behavioral change, just as attitudes are crucial aspects of behavioral prediction models. Furthermore, exercise behavior can facilitate adherence. Three mechanisms are involved and are highly interrelated. First, by focusing on the immediate purposes of exercise, motivation during exercise promotes effort-based decision-making and the involvement of people in the session. Second, repeated exercise sessions can lead to increases in executive control, thereby improving individuals’ ability to inhibit bad habits and to plan future exercise. Third, exercise behavior that involves effort can help individuals change their attitude by reducing cognitive dissonance. Thus, it is necessary not only to involve individuals in long-term practice by developing their motivation and willpower but also to help counselors increase an individual’s level of commitment to a behavior by using preparatory acts or behavioral situations that will reduce dissonance in favor of the target behavior. Because exercise adherence refers to the strength of the link between attitudes and behavior, it is important that attitudes are involved when such behavior occurs. In other words, repetition of a short cycle of exercise leads to long-term modification of behavior by reinforcing the link between attitude and behavior.

Concomitant effects may be observed between these three pathways. Cognitive dissonance occurs when there is a discrepancy between a person’s beliefs and behavior. This can occur when people act in a way that is inconsistent with the way they believe they should act. According to cognitive dissonance theory, people should work to reduce dissonance, and if they are involved in an exercise session (with immediate purpose, such as graded tasks or digital tools showing their progress over time), they may modify their beliefs to account for or justify their behavior (effort justification). Put another way, the more difficult the task is, the greater the value given to the reinforcers that follow task completion. The phenomenon of attributing added value to reinforcers based on the effort of a task enables changes in attitude toward the behavior performed (e.g., exercising). For example, dissonance is already present among people with low levels of motivation to exercise. To reduce this dissonance, people should be involved in the exercise at an effort level that allows them to modify their attitude to justify effort involvement. If the level of effort is too low, the immediate purpose (the reinforcer) will not be valued, and the dissonance will be reduced in the sense of the initial low motivation. Therefore, immediate purpose is both a motivation for action and a lever for dissonance reduction. Thus, by strengthening the link between attitude and behavior (i.e., by increasing adherence), people will be able to deploy more effort and to involve more executive control. In contrast, when people are motivated to be involved in exercise sessions, the dissonance is low or nonexistent, and it could be important to use preparatory acts, such as sponsoring a peer in a free manner, to increase commitment to the behavior and strengthen the link between attitude and behavior.

Nevertheless, these three pathways can also collide. For instance, external rewards, such as social support, are viewed as useful for facilitating motivation but can exert deleterious effects on the dissonance reduction process. Indeed, when external rewards justify the level of effort deployed during the task (e.g., to please someone), people will consider the reward as justifying the level of effort and will not change their attitude by evoking external causes, thereby reducing adherence. To avoid this negative effect, it may be necessary to target internal rewards, such as positive mood or body change, to reduce dissonance by changing attitudes. When people are motivated, it is possible not to offer systematic rewards or to offer low external rewards after evoking freedom to solicit internal reasons.

Altering the paradigm of behavioral change models seems to be a novel approach for promoting exercise adherence. The target behavior (exercise) also facilitates change not only by developing motivation and volition but also by changing attitudes. In other words, involving and committing people in their exercise behaviors is an effective lever for initiating and developing adherence, and behavioral change theories could benefit from this behavioral perspective.

## Conclusion

Behavioral change is a complex process. Durable behavioral changes must be accompanied by durable changes in attitudes. This model mainly applies to behaviors that require effort to be carried out, such as starting a good habit or quitting a bad habit. As part of the exercise, it is always possible to consider the activity level of the individual, which can provide an interesting starting point. Indeed, no one is completely inactive. People still perform behaviors such as going shopping or walking to get from one place to another. This aspect can be a central element in creating dissonance and helping to reduce it in the direction of the targeted behavior. However, further research is needed to determine when and how these three processes interact with one another.

## Data Availability

Not applicable.
